# Why do babies cry? Exploring the role of the gut microbiota in infantile colic, constipation, and cramps in the KOALA birth cohort study

**DOI:** 10.1080/19490976.2025.2485326

**Published:** 2025-03-30

**Authors:** David Barnett, Carel Thijs, Monique Mommers, Martha Endika, Cynthia Klostermann, Henk Schols, Hauke Smidt, Arjen Nauta, Ilja Arts, John Penders

**Affiliations:** a Maastricht Centre for Systems Biology, Maastricht University, Maastricht, Netherlands; b NUTRIM School for Nutrition and Translational Research in Metabolism, Department of Medical Microbiology, Infectious Diseases and Infection Prevention, Maastricht University Medical Center+, Maastricht, Netherlands; c CAPHRI Care and Public Health Research Institute, Department of Epidemiology, Maastricht University, Maastricht, Netherlands; d Laboratory of Microbiology, Wageningen University & Research, Wageningen, Netherlands; e Department of Food Chemistry, Wageningen University & Research, Wageningen, Netherlands; f FrieslandCampina Ingredients, FrieslandCampina, Amersfoort, Netherlands

**Keywords:** Infant gut microbiota, functional gastrointestinal symptoms, colic, constipation, human milk oligosaccharides

## Abstract

Gastrointestinal symptoms are common during infancy, including infantile colic. Colic can be loosely defined as prolonged and recurrent crying without obvious cause. The cause indeed remains unclear despite much research. Results on infant nutrition are inconclusive, but prior work has linked maternal mental health to infant crying. Recently, several small studies have described associations between gut microbiota and colic. We used a larger cohort to examine the role of the microbiota in infant gastrointestinal health, while also accounting for other biopsychosocial factors. Using fecal 16S rRNA gene amplicon sequencing data from 1,012 infants in the KOALA birth cohort, we examined associations between the 1-month gut microbiota and parent-reported functional gastrointestinal symptoms throughout infancy, including colic, constipation, and cramps. These analyses were adjusted for biopsychosocial factors that were associated with symptoms in a broader analysis involving 2,665 participants. In 257 infants, we also explored associations between breastmilk human milk oligosaccharides (HMOs) and gastrointestinal symptoms. Higher relative abundance of *Staphylococcus* at one month was associated with less constipation in the first three months of life. Conversely, *Ruminococcus gnavus* group abundance was associated with more colicky symptoms, particularly between four and seven months. Breastmilk concentrations of the HMOs lacto-N-hexaose (LNH) and lacto-N-neohexaose (LNnH) were associated with less constipation in the first three months. Our results support the conclusion that gut microbiota are relevant in infantile colic and constipation. However more work is needed to elucidate the underlying mechanisms, and explore their interplay with other relevant biopsychosocial factors such as maternal mental health.

## Importance

During infancy, functional gastrointestinal symptoms such as colic, constipation, and cramps are common and burdensome, affecting both infants and their caregivers. The underlying causes of these symptoms, particularly colic, remain poorly understood, hindering the development of effective treatments and preventive strategies.

In our large-scale study involving over 1,000 Dutch families, we report associations between infant gut microbiota and gastrointestinal symptoms. We identify high *Ruminococcus gnavus* relative abundance as a risk factor for colicky crying. Given that *R. gnavus* has been linked to the pathophysiology of irritable bowel syndrome and inflammatory bowel diseases in adults, our findings suggest that some cases of colic may share similar pathophysiological mechanisms. Additionally, within a subgroup of breastfed infants, we observed that higher concentrations of human milk oligosaccharides LNH and LNnH in breastmilk were associated with lower risk of constipation. Exploring these oligosaccharides further could lead to effective intervention strategies.

## Introduction

Parents often observe the signs of various functional gastrointestinal disorders in their infants, including constipation, regurgitation, cramps, and colic.^1^ Colic is a common problem in the first three or four months of life, defined loosely as recurrent and prolonged crying, with no obvious cause.^2,3^ Parents may report visible abdominal cramps as part of a colicky presentation, but this is not a sign exclusive to colic. Constipation in infants can be recognized by large or hard stools, with infrequent or painful defecation.^4^ Despite being common, and largely self-limiting, colic, constipation, and other gastrointestinal symptoms can substantially impair the wellbeing of infants and carers, particularly if the symptoms are unusually persistent.^5^

The pathophysiology of most functional gastrointestinal symptoms remains quite enigmatic, despite decades of research, particularly around colic.^6,7^ Recently, there has been increasing interest in the hypothesis that the infant gut microbiota plays a role in gastrointestinal symptom pathophysiology, which seems plausible for several reasons. A handful of small prior studies have identified associations between the infant gut microbiota and infantile colic and constipation symptoms.^8–12^ Clinical trials aimed at colic symptom reduction by probiotic supplementation, mostly lactobacilli, have shown mixed results.^13–17^ In addition, numerous studies have demonstrated that the gut microbiota of older children and adults with irritable bowel syndrome differs from the microbiota of healthy controls.^18^ Hypothesized mechanisms for causal links between gut microbes and gastrointestinal discomfort include their roles in gas production and inflammation.^19,20^ More generally, there is a large body of literature reporting associations between infant gut microbiota composition and subsequent child health status, including atopic disease risk, growth, metabolism and neurodevelopment, as well as demonstrated mechanistic links between the infant microbiota and immune system maturation, inflammation homeostasis, and nutrient metabolism.^21–25^

Preceding the recent interest in the gut microbiota, substantial research efforts have investigated the role of other biopsychosocial factors in infant gastrointestinal symptom pathophysiology, including maternal mental health and infant feeding. Maternal mental health has repeatedly been associated with infant colic, likely representing a multifaceted bidirectional relationship.^26–29^ Breastfeeding duration has been associated with colic risk, but quite inconsistently across studies, leaving breastmilk’s role in colic unclear.^30–32^ Breastfeeding appears to support healthy stool consistency and frequency during infancy,^33^ with some speculation that human milk oligosaccharides (HMOs) in breastmilk mediate some part of these benefits.^34^ HMOs are a diverse collection of non-digestible carbohydrates that can beneficially influence microbiota development in breastfed infants.^35^

The role of microbiota should be considered within the broader context of these other biopsychosocial factors, particularly given the complex interplay between maternal wellbeing and breastfeeding efficacy and choices,^36–38^ and the profound effect that breastfeeding has on the infant gut microbiota composition.^39–42^

In this article we first present an exploratory analysis of various biopsychosocial risk factors for infant colic, cramps, and constipation using a large dataset on over 2,600 mother-infant dyads from the KOALA birth cohort.

The primary focus of this article is then centered around a subset of over 1,000 infants with fecal microbiota data. In this group we examine microbiota associations with gastrointestinal symptoms, and assess whether these associations may be confounded by other biopsychosocial risk factors from our exploratory analyses, such as maternal distress and infant feeding differences.

In addition, we collected breastmilk samples from a subset of over 250 mother-infant pairs, allowing us to assess the contribution of natural variation in HMO content to functional gastrointestinal symptom risk amongst breastfed infants.

## Methods

### KOALA study design

The KOALA Birth Cohort Study is a prospective birth cohort in the Netherlands, approved by the Ethics Committee of Maastricht University Medical Center. The cohort design has been described in detail elsewhere.^40,43^ The study recruited 2,834 pregnant women, using two strategies: most women were enrolled from an ongoing study investigating pregnancy-related pelvic girdle pain (the conventional recruitment group); and a further group of pregnant women were recruited through advertisements placed in locations associated with “alternative” lifestyle choices, such as organic shops, anthroposophic clinics and Steiner schools (the alternative recruitment group). All parents provided informed consent before participation. For the analyses in this manuscript, we excluded the second-born of twin pairs, infants born preterm (<36 weeks), and children with significant genetic, congenital, or neurodevelopmental disorders.

### Gastrointestinal symptoms: data collection and operationalisation

Mothers received questionnaires at three, seven, and twelve months postpartum. They were asked to report the frequency and duration of crying, cramps, and constipation symptoms observed in their infant during the preceding three months, or since the last questionnaire.

We used the Modified Wessel’s criteria^3^ to define crying symptoms as infantile colic symptoms, namely the report of crying for over three hours per day, for over three days in one week. We defined similar criteria for persistent cramp symptoms, and for persistent constipation symptoms: the symptoms must be reported to be present during at least three days in one week.

### Maternal distress measurement

Mothers were asked to complete the 12-item General Health Questionnaire (GHQ-12) at approximately two weeks postpartum.^44^ From the 3-point Likert scores of the 12 items we computed a continuous 36-point distress score for each mother, and applied z-score standardization before statistical modeling.

### Other biopsychosocial questionnaire data

From the parent questionnaires completed during pregnancy and the first year postpartum, we also obtained information on: infant sex (male/female); maternal age (years); maternal education level (higher/lower), maternal history of irritable bowel syndrome (yes/no); KOALA recruitment group (conventional/alternative); birth mode (C-section/vaginal); birth place (home/hospital); presence of older siblings (yes/no); presence of pet cats (yes/no); presence of pet dogs (yes/no); infant antibiotic exposure during the first three months of life (yes/no); maternal antibiotic exposure during pregnancy (yes/no); tobacco smoking during third trimester (yes/no); alcohol consumption during third trimester (yes/no); and breastfeeding history (ever/never breastfed). We used breastfeeding initiation and not breastfeeding duration because feeding mode changes could be affected by early infant crying and gastrointestinal symptoms, and would thus introduce a risk of confounding by reverse causation.

### Microbiota data generation and processing

The KOALA study collected 1,176 fecal samples from infants at around one-month of age, starting collection halfway through the study’s recruitment period. Parents collected a fecal sample from a sanitary napkin placed in the infant’s diaper and mailed the collection tube to Maastricht University Medical Center the same day. Samples collected <21 days or >49 days postpartum were excluded from analysis. Fecal DNA was isolated by repeated bead beating and column-based purification using the QIAamp DNA stool mini kit (Qiagen, Hilden, Germany) as described previously.^45^

Amplicon libraries were generated by PCR amplification using barcoded primer pairs targeting the 16S rRNA V4 variable gene region (515F-806 R).^46,47^ Amplifications were performed in triplicate, using 25 cycles for reactions containing 1.4-20ng template DNA or 30 cycles for samples with < 1.4 ng DNA. Libraries were sequenced using paired-end Illumina HiSeq sequencing (Eurofins Genomics, Germany), including mock communities and no-template controls.

Amplicon sequence variants (ASVs) were inferred using NG-Tax2^48^ with default settings, and trimming the forward and reverse reads to the length of 80 bases. Taxonomic annotation of ASVs was achieved using the SILVA-132 reference database^49^ and a phylogenetic tree was constructed using phangorn.^50^ Reads assigned to the genera *Ralstonia* or *Cupriavidus* were removed from the dataset prior to statistical analyses, as these were consistently detected in our negative control data and are known to commonly contaminate lab kits. If an ASV could not be uniquely classified at a particular rank, they were aggregated together for the taxonomically-aggregated statistical analyses under the name of the lowest classified rank, e.g. “*Enterobacteriaceae* family”. Samples with fewer than 15,000 taxonomically-classified reads were considered low-quality samples and excluded from further analysis.

### HMO data generation and processing

317 breastfeeding mothers provided breastmilk samples approximately one month after delivery, which were collected into sterile tubes, refrigerated, and transported on ice to Maastricht University Medical Center that day.^46^

Samples were stored at −80°C and HMO profiles for 15 distinct structures were obtained using high performance liquid chromatography and mass spectrometry, as described in detail in the supplementary methods and elsewhere.^46^ We obtained measurements for seven fucosylated structures (2′-FL, DFL, LNDFHI, LNFPI, LNFPII, LNFPIII, and LNFPV), three non-fucosylated neutral structures (LNH, LNnH, and a combination of LNT and LNnT referred to as LN(n)T) and five sialylated structures (3´-SL, 6´-SL, LSTa, LSTb and LSTc).

### Statistical analyses

We performed data handling, statistical analyses, and visualizations with R (version 4.3.2), the Tidyverse packages^51^ and the seriation package.^52^ We calculated 95% confidence intervals with the Profile Likelihood method and used an alpha level threshold of 0.05 for statistical significance. We used a complete-case analysis approach, excluding participants on a per-analysis basis if the relevant exposure or outcome data were missing. We performed multiple-testing corrections by using the Benjamini-Hochberg false discovery rate (FDR) correction method, on p-values grouped by outcome at each age.

#### Crude biopsychosocial associations

We fitted multiple separate single-predictor binary logistic regression models to estimate each of the crude statistical associations between each explanatory variable and each binary indicator variable for each symptom within each age range (0–3 months, 4–7 months, and 8–12 months).

#### Microbiota associations

We used the R packages phyloseq 1.44.0, vegan 2.6.4, and microViz 0.12.1 for the microbiota analyses.^53–56^

**Figure 1.**
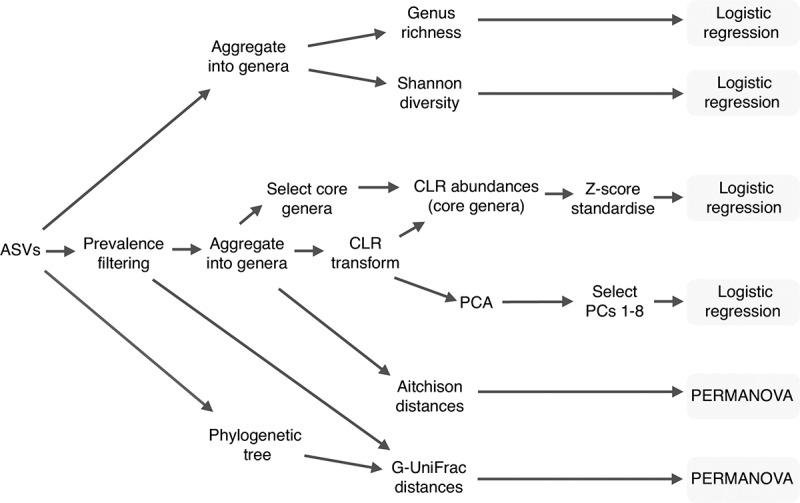
Diagram illustrating operationalisation of the microbiota sequencing data for statistical analyses.

We used four complementary approaches to assess associations between the one-month gut microbiota composition and each reported gastrointestinal symptom within each age range (Figure 1): permutational multivariate analysis of variance (PERMANOVA); alpha diversity regression models; core genera regression models; and principal component (PC) regression models. To reduce noise arising from errors in data generation we filtered out rare ASVs (<2.5% prevalence) prior to all analyses except for the alpha diversity calculations.

Firstly, to assess crude associations between overall microbiota composition and each symptom at each age, we applied PERMANOVA using the vegan function adonis2 with 9,999 free permutations. As our main dissimilarity measure, we used Aitchison distances computed with read counts aggregated at the level of genus. As a secondary dissimilarity measure we used GUniFrac 1.8 to compute the Generalised UniFrac (G-UniFrac) distance with default ASV abundance weighting, using a phylogenetic tree inferred from our ASV sequences with phangorn 2.6.2 as described in Callahan et al. 2016.^50,57^

To ascertain which aspects of microbiota composition may be associated with each symptom, we used logistic regression models. Each model included one binary outcome variable and one microbiota-derived predictor variable. We fitted crude models and covariate-adjusted models, which included the biopsychosocial predictors of gastrointestinal symptoms that were supported by the previous crude biopsychosocial association analyses.

As predictor variables in the alpha diversity regression models, we used either the genus-level observed richness or the genus-level Shannon diversity index. As predictor variables in the core genera regression models we used centered log ratio (CLR) transformed and z-score standardized abundance of each core genus. We defined core genera as any genus-level feature that reached at least 1% relative abundance in at least 10% of infants’ samples. CLR transformation was applied to address the compositionality of the sequencing data,^58^ and zeroes were replaced with a pseudocount of half the overall minimum observed abundance. Z-score standardization was applied to facilitate visual and statistical comparison across genera.

We hypothesized that (subsequent) antibiotic exposure may reduce any effects of one-month microbiota composition on gastrointestinal pathophysiology, and so we also fitted the core genera regression models again but excluding infants with reported antibiotic exposure in the first three months of life, as a sensitivity analysis. Furthermore, to aid comparison with prior studies of the breastfed infant microbiota and gastrointestinal symptoms, we also fitted the core genera regression models to only the infants exclusively breastfed prior to the fecal sample microbiota profiling.

Finally, we used PC regression models as an alternative approach to test for further patterns of microbiota variation that may be associated with infant symptoms. To obtain the principal components used as predictor variables in these models, we applied principal components analysis (PCA) to the full CLR-transformed genus-level count matrix.

#### HMO associations

The estimated breastmilk HMO concentrations were converted to proportional abundances by dividing each concentration by the sample’s arbitrary total concentration. To correct for the often moderately right-skewed HMO abundances we used a square root transformation prior to statistical modeling. We fit separate binary logistic regression models for each HMO and outcome combination per age range, adjusted for HMO measurement batch. We did not fit models for outcomes that were reported for fewer than 10 infants during a given age range in this data subset.

## Results

### Study population

**Figure 2.**
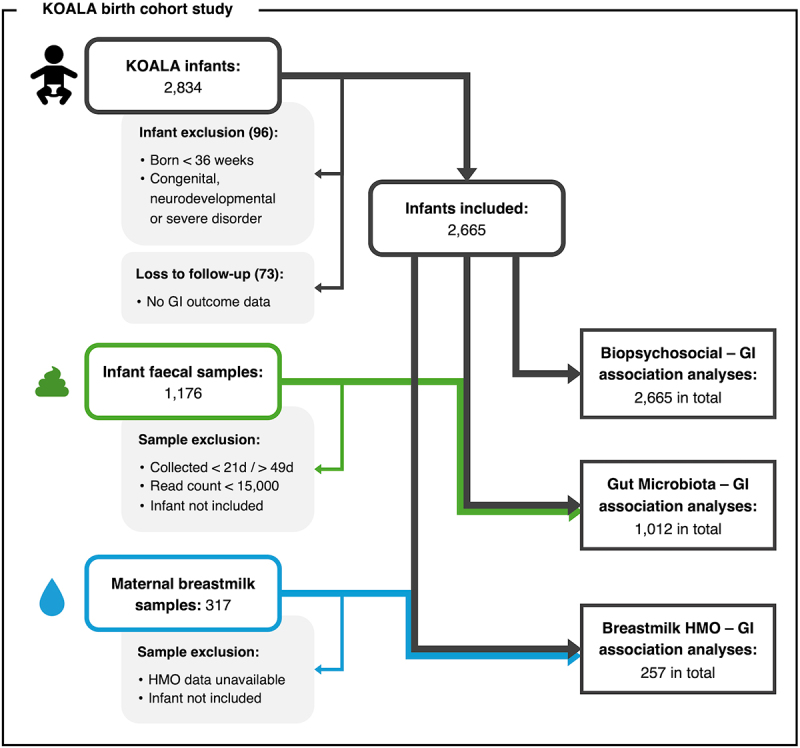
Flowchart illustrating study population and overview of analyses.

A total of 2,665 mother-infant dyads were included in our analyses of biopsychosocial risk factors for infant functional gastrointestinal symptoms (Figure 2). The mothers’ median age was 32 years (IQR 30–34) and 47.9% reported a higher level of education. Alcohol consumption and smoking during the third trimester of pregnancy were reported by 437 and 181 mothers respectively (16.4% and 6.8%). Antibiotic exposure during pregnancy was reported by 405 mothers (15.2%), and 171 infants had received antibiotics by 3 months of age (6.7%).

Many infants were born at home (45.3%), and 292 were born by C-section (11.0%). Around half of the infants had older siblings (56.2%) and the vast majority received some breastfeeding (84.2%).

Our analyses of the associations between neonatal microbiota composition and functional gastrointestinal symptoms included 1,012 of these infants, those with fecal microbiota data. Our analyses of the associations between maternal breastmilk HMO profile and symptoms included 257 breastfed infants for whom we had HMO data. The population characteristics of these microbiota data and HMO data subgroups were comparable to the larger cohort, except that a larger proportion of mothers reported a higher education level in these subgroups (Supplementary Table S1).

### Symptom patterns

**Figure 3.**
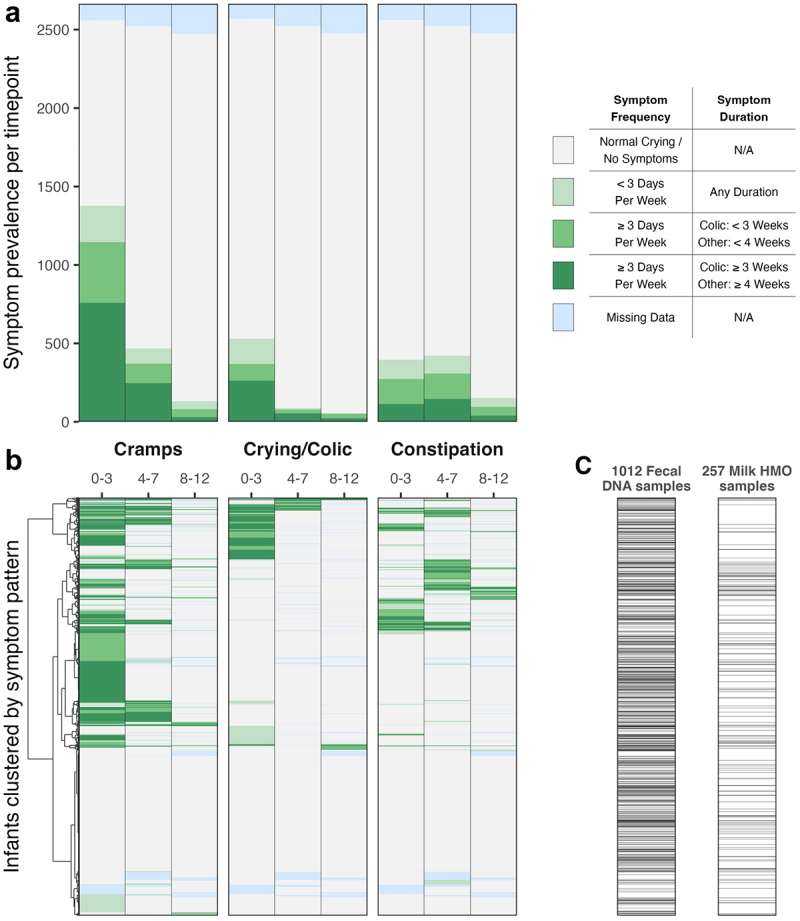
Infant gastrointestinal symptom frequencies and co-occurrence patterns (*N* = 2665) (a) Stacked bar chart of the prevalence of each symptom within each infant age window, with colour shade indicating the level of symptom persistence. (b) Heatmap illustrating the patterns of symptom cooccurrence within infants. Infants are sorted by hierarchical clustering of their symptom profiles. The four symptom levels are encoded as digits one through four, with missing values imputed with the mean for that symptom at that age. Hierarchical clustering was performed with the Ward method using Euclidean distances computed on the square root of each numeric symptom profile. (c) Strip plots for the availability (grey) or absence (white) of each biological sample data type for each infant.

Functional gastrointestinal symptoms were common, with 1,788 out of 2,665 children (67.1%) having at least one symptom reported during infancy (Figure 3a, Supplementary Table S2). Symptoms were weakly positively correlated with each other, particularly in early infancy (Supplementary figure S1).

Cramps was the most common symptom, particularly in early infancy. In their first three months of life, 1377 infants (53.8%) were reported to have experienced at least some cramps, and 1146 infants (44.8%) met our definition of persistent cramps, by having at least three symptomatic days within a week. Only 22.9% of these also displayed persistent crying symptoms (Figure 3b, 262 infants). Between four and seven months of age, 371 infants (14.7%) were reported to have had persistent cramps symptoms. 80.6% of these were infants who already showed earlier persistent cramps (299 infants).

Crying symptoms indicative of infantile colic (≥3 hours in a day) were reported in 530 infants up to three months of age (20.6%). 369 (14.4%) infants met our working definition of infantile colic, displaying at least three hours of crying for at least three days in a week (i.e. Modified Wessel’s Criteria), and 263 of these cases persisted for at least three weeks in the first three months of life (10.2%). 71% of the 369 colicky infants also displayed persistent cramps (262 infants). Colicky crying symptoms were much less common after three months, with 79 cases reported between 4 and 7 months (3.1%), of whom 47 were also earlier cases.

Persistent constipation was the only symptom to be reported in similarly frequency in both the 0- to 3-month and 4- to 7-month age ranges, affecting 273 (10.7%) and 308 (12.2%) infants respectively. However, infants were rarely symptomatic with constipation in both age ranges (89 infants, 3.3%).

#### Biopsychosocial associations with infant gastrointestinal symptoms

**Figure 4.**
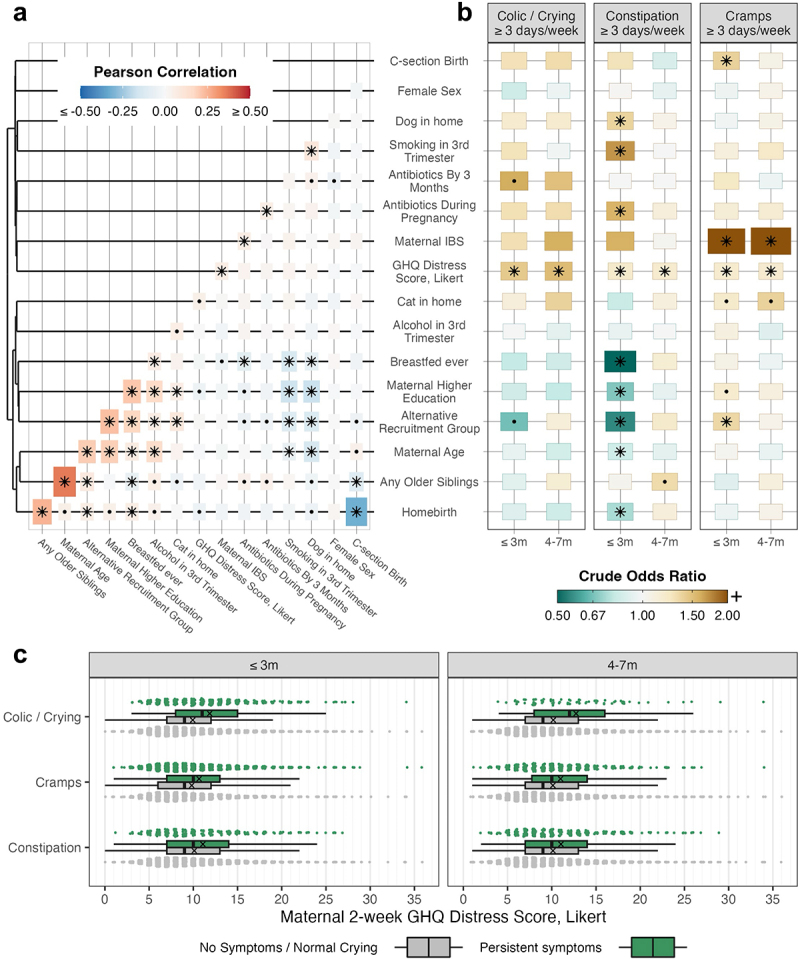
Biopsychosocial factor intercorrelations and crude associations with infant gastrointestinal symptoms (*N* = 2665) a) Heatmap of Pearson correlations between pairs of biopsychosocial factors. Heatmap is sorted with hierarchical clustering with optimal leaf ordering based on the Ward method using Euclidean distances between correlation profiles. Dots indicate *p* < 0.05 and asterisks indicate FDR < 0.05 (corrected per predictor). Tile colour indicates the correlation coefficient, with extreme values limited to ±0.5. Tile size is proportional to the absolute value of the correlation coefficient. b) Heatmap illustrating the direction and strength of crude associations between each biopsychosocial predictor variable and each gastrointestinal outcome. Dots indicate *p* < 0.05 and asterisks indicate FDR < 0.05 (corrected per outcome per timepoint). Tile colour indicates the crude odds ratio, with values ≥2 capped at the upper limit of the colour scale. Tile size is proportional to the absolute value of the regression coefficient. All values are available in supplementary table 3. c) Boxplots and Sina jitterplots displaying the distribution of maternal distress scores according to infant symptom status per symptom per age window. The “X” indicates the mean score for that group. FDR: False Discovery Rate.

Many of the biopsychosocial factors were crudely associated with at least one gastrointestinal symptom, particularly in early infancy (Supplementary Table S3). Several inter-correlated maternal characteristics and behaviors were associated with lower constipation risk in the first three months (FDR <0.05) including higher maternal age, higher education level, and ever breastfeeding (Figure 4a,b). Conversely, third trimester smoking and pregnancy antibiotics exposure were both associated with increased constipation risk (FDR <0.05).

Maternal distress, as reported at two weeks post-postpartum on a GHQ-12 questionnaire, was significantly associated with an increased risk of every symptom at all timepoints and was not correlated with any of the other predictors. Maternal distress scores varied widely, but mothers of infants with any reported symptom during infancy had on average 1 to 3 points higher total distress score than mothers of infants without that symptom (Figure 4c).

All the biopsychosocial factors that were associated with at least one symptom at one timepoint were selected to be covariates in microbiota-gastrointestinal models.

#### Neonatal microbiota associations with infant gastrointestinal symptoms

According to the results of crude PERMANOVA analyses with genus-level Aitchison distances, overall one-month microbiota composition was significantly associated with infantile colic (*p* = 0.013, 142 cases, *N* = 999) and persistent constipation symptoms (*p* = 0.018, 101 cases, *N* = 993) in the first three months but not with persistent cramps (*p* = 0.650, 479 cases, *N* = 996), or with any of the symptoms at later ages (Supplementary Table S4a). A similar pattern was observed in our alternative PERMANOVA models using GUniFrac distances, although no association reached statistical significance (Supplementary Table S4B). Overall genus richness and Shannon diversity were not significantly associated with any symptom at any age after multiple testing corrections (Supplementary Table S5).

**Figure 5.**
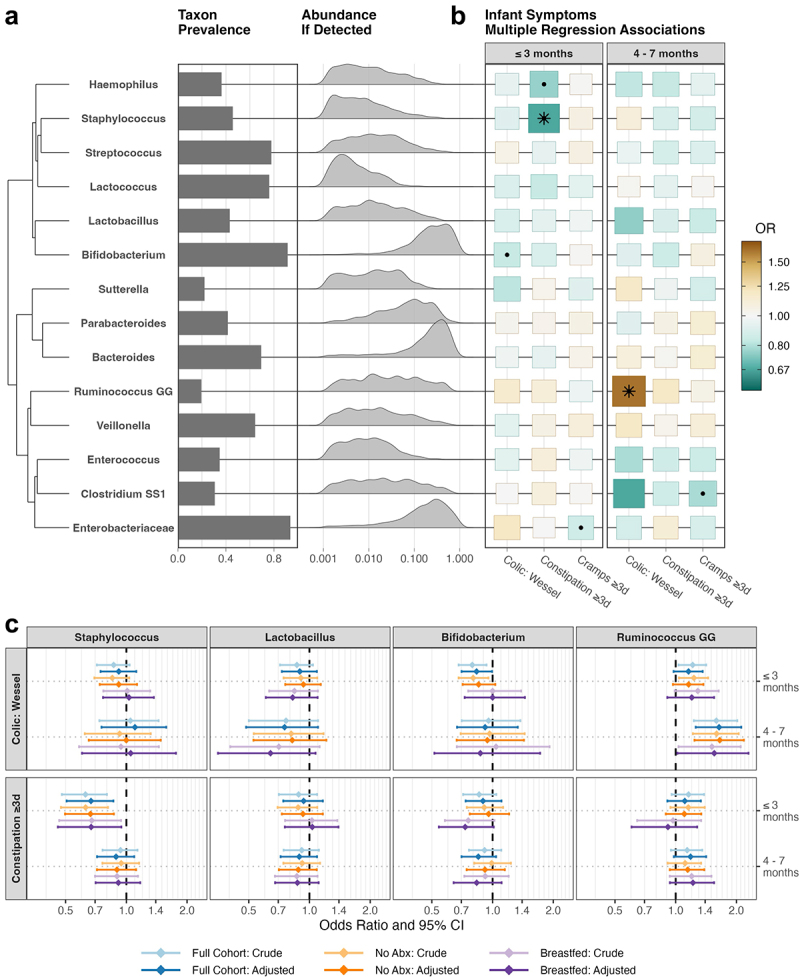
Infant gut microbiota core genera distributions (*N* = 1012) and associations with gastrointestinal symptoms (a) Bar charts of taxa prevalence and frequency density ridge plots of relative abundance of each taxon within the subset of infant samples in which it was detected (i.e., omitting zeros). Taxa are sorted by hierarchical clustering and optimal leaf ordering using the Ward method on Euclidean distances between their normalised clr-transformed abundance profiles. (b) Heatmap illustrating the direction and strength of covariate-adjusted associations between each genus and each gastrointestinal outcome. Dots indicate *p* < 0.05 and asterisks indicate FDR < 0.05 (corrected per outcome). Tile colour indicates the odds ratio for a one standard deviation increase in CLR-transformed abundance of that genus. Tile size is proportional to the absolute value of the regression coefficient. A full list of covariates is included in Supplementary Table S6. (c) Point-range Forest plots illustrating the logistic regression association odds ratios and 95% confidence intervals for crude and covariate-adjusted models in the full cohort of infants with microbiota data and in the subset of infants not exposed to antibiotics in the first three months of life, and in the subset of infants exclusively breastfed until fecal sampling at one month of age. CLR: Centered Log Ratio. FDR: False Discovery Rate.

We identified 14 core genera within our cohort of 1,012 infants with one-month fecal microbiota data, defined as reaching at least 1% relative abundance in at least 10% of infants’ samples (Figure 5a). Many infants’ microbial ecosystems were predominated by *Bifidobacterium*, *Bacteroides*, or genera within the *Enterobacteriaceae* family. Upon hierarchical clustering of the patterns of normalized CLR-transformed abundances, these three predominant taxonomic groups cluster separately, each with other core genera of lower prevalence or abundance.

We observed interesting associations between several genera and infant gastrointestinal symptom reports, which persisted after statistical adjustment for the other biopsychosocial determinants identified earlier (Figure 5b,c, Supplementary Table S6).

Higher relative abundance of *Staphylococcus* was associated with significantly lower risk of persistent constipation in the first three months (Figure 3b, FDR = 0.042, 100 cases, *N* = 973). There was also some evidence for this association in infants who were exclusively breastfed prior to fecal sample collection (*p* = 0.022, FDR = 0.338, 41 cases, *N* = 555).

*Haemophilus* was also associated with lower risk of constipation in the first three months of life (*p* = 0.034), but not significantly after FDR correction (FDR = 0.301). A higher relative abundance of *Bifidobacterium* was associated with a lower risk of infantile colic in the first three months (*p* = 0.043, 139 cases, *N* = 978), but this association was not significant after FDR correction (FDR = 0.321) and did not hold in the exclusively breastfed infants (*p* = 0.999, 53 cases, *N* = 557). Conversely, there was some evidence that higher relative abundance of *Bifidobacterium* may be associated with a lower risk of constipation only amongst the breastfed infants (*p* = 0.060, FDR = 0.338). A higher relative abundance of *Ruminococcus gnavus* group (*Ruminococcus* GG) was significantly associated with a higher risk of colicky symptoms between four and seven months of age (FDR = 0.002, 33 cases, *N* = 964) with a similar but non-significant association in the first three months of life (*p* = 0.090).

Principal components regression analyses on CLR-transformed genera, demonstrated approximately similar associations. In addition, these analyses highlighted a possible connection between high *Lactobacillus* and fewer colicky symptoms, particularly between four and seven months of age, as *Lactobacillus* was heavily loaded on the fifth principal component, which was associated with a lower risk of colic symptoms in that age window (FDR = 0.002, Supplementary Figure S2, Supplementary Table S7).

#### Breastmilk HMO profiles and early infant gastrointestinal symptoms

**Figure 6.**
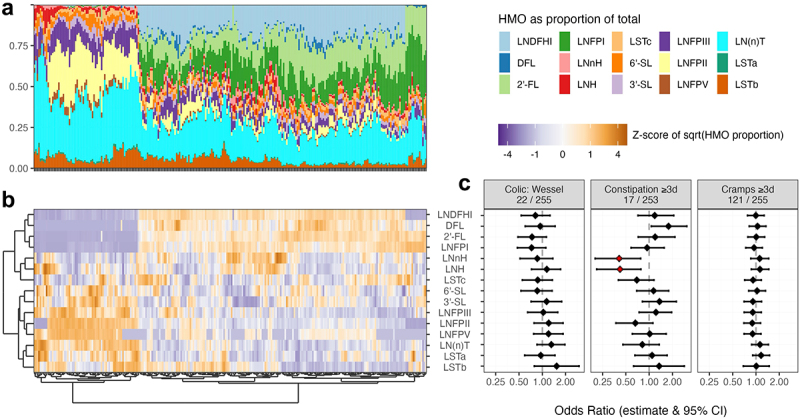
Breastmilk HMO profiles (*N* = 257) and associations with gastrointestinal symptoms up to three months of age. (a) Stacked bar chart of proportional HMO profiles for each mother’s milk sample, sorted to match the heatmap. (b) Heatmap of breastmilk HMO profiles displaying the normalised square root of each HMO’s proportional abundance and with samples and features sorted using hierarchical clustering with the Ward method and optimal leaf ordering. (c) Point-range Forest plots illustrating the logistic regression association odds ratio point estimates and 95% confidence intervals for a one standard deviation increase of that HMO’s square root transformed proportional abundance and symptom during the first three months of life. Number of cases/complete observations per analysis are listed under each symptom. These models are adjusted for HMO measurement batch. The red asterisks indicate significance, FDR < 0.05 (corrected per outcome). FDR: False Discovery Rate.

We profiled 257 mothers’ milk samples for 15 distinct HMO structures (Figure 6a). The compositions of the milk samples clustered neatly into four categories representing maternal Secretor status and Lewis group combinations, using hierarchical clustering with the Ward method and optimal leaf ordering on the normalized square root of each HMO’s proportional abundance (Figure 6b).

Higher proportional abundances of each of the HMOs LNH and LNnH were significantly associated with a lower risk of persistent constipation symptoms in the first three months of life (Figure 6c, logistic regression models, measurement batch adjusted, FDR < 0.05, Supplementary Table S8). Both LNH and LNnH are relatively low abundance neutral HMOs, and their normalized profiles cluster together on hierarchical clustering (Figure 6b).

None of the HMOs were associated with persistent constipation between four and seven months, nor with symptoms between eight and twelve months. Infantile colic in the first three months of life was not associated with any HMO, and colicky symptoms were too uncommon in later infancy (<7 cases) to allow statistical analysis in this subgroup of our cohort. Persistent cramps symptoms were not associated with any of the HMOs at any age in infancy.

## Discussion

Through a large-scale observational analysis, our findings support the emerging hypothesis that the infant gut microbiome is a relevant factor in the development of infantile colic and constipation symptoms. Infants who never received breastmilk were more often constipated, and breastfed infants receiving higher levels of the neutral HMOs LNH or LNnH were less often constipated than other breastfed infants.

In our analyses of 1,012 infants, we observed associations between the overall one-month gut microbiota composition and both colic and constipation symptoms during the first three months of life. In particular, we identified two associations with strong statistical support in regression models adjusted for numerous other biopsychosocial factors and after applying multiple testing corrections: an association between *Staphylococcus* and less constipation, and an association between *Ruminococcus gnavus* group and more colicky crying symptoms later in infancy.

A higher relative abundance of *Staphylococcus* corresponded with less constipation during the first three months of life. *Staphylococcus* is a typical skin surface resident, also found in breastmilk, and infants may ingest it during breastfeeding, for example.^59,60^ We have previously shown that breastfed neonates have higher relative abundance of *Staphylococcus*,^42^ and in this work we found that infants who were never breastfed had higher rates of constipation. However, we observed that the association between *Staphylococcus* and lower constipation also persists amongst infants who were exclusively breastfed prior to microbiota profiling, which suggests that high *Staphylococcus* is not just an indicator of breastfeeding history. In addition, it may be interesting to note this negative association is significant in both the crude and covariate-adjusted analyses, despite the tendency for C-section birth (a potential confounder) to be associated positively both with *Staphylococcus* ^42^ and constipation. Moreover, gastrointestinal transit time is known to be strongly related to the measured fecal microbiota profile, at least in adults, and so it is not impossible that early constipation itself causes the altered infant fecal microbiota composition we observe.^61^ There are little prospective data on constipation during infancy, with most research instead focusing on established childhood constipation cases. In Korpela et al. 2020, they did not find any significant associations between the meconium microbiota and infant constipation, but the average relative abundance of *Staphylococcus* in neonates with later constipation was half that of those without later constipation. The direction of association concurs with our results and supports the possibility that low *Staphylococcus* might precede constipation.^10^ Another study compared qPCR counts of total bacteria, *Lactobacillus* and *Bifidobacterium* in 39 constipated infants and children (6 months to 3 years old) with 40 age-matched controls.^9^ They found a lower level of *Lactobacillus* in constipated participants, however the comparison was not adjusted for the much shorter average breastfeeding duration in constipated children. We found no evidence for an association between lower *Lactobacillus* and infant constipation in our analyses, but we did note a non-significant (*p* = 0.06) association between lower *Bifidobacterium* and higher constipation risk amongst exclusively breastfed infants in their first three months.

Colic has been more extensively studied in several smaller microbiota-focused projects which overall show a limited degree of consistency with each other and with our findings, and overall support the general premise that the gut microbiota is involved in colic pathogenesis. Discrepancies may be explained by the heterogeneity in study population characteristics (e.g. breastfeeding rates); microbiota profiling methods (16S rRNA gene amplicon sequencing, qPCR, and various other techniques); ages of fecal sampling (a range from the first days of life^10^ to several months after birth);^62,63^ and statistical approaches. We compare our findings to these studies below, but we also refer readers to the reviews of Johnson and Adams,^8^ and Hofman and colleagues^11^ for a comprehensive overview of these studies. A more recent study of almost 700 infants in the Danish COPSAC_2010_ cohort concluded that 16S rRNA gene amplicon sequencing derived profiles of the gut microbiota at one month were weakly predictive of colic up three months of age, but they did not detect statistically significant associations with specific genera.^64^

We observed that *Ruminococcus gnavus* group abundance at one month was associated with a significantly higher risk of colic symptoms in the four-to-seven-month age range, as well as showing weak evidence for a similar relationship in the first three months. Although commonly found in the human gut microbiota throughout life, high relative abundance of *Ruminococcus gnavus* has been repeatedly associated with irritable bowel syndrome (IBS) and inflammatory bowel disease symptoms in adults, and it is known to produce an inflammatory polysaccharide.^18,65–67^ We thus speculate that a high relative abundance of *Ruminococcus gnavus* may play a role in inflammatory pathophysiology in some infants with colicky symptoms. Differences in *Ruminococcus gnavus* abundance have not been noted in prior studies of infantile colic. It is plausible that this discrepancy can be explained by our larger sample size, which allows greater statistical power to detect associations with less common taxa such as the *Ruminococcus gnavus* group. *Ruminococcus gnavus* appears to have been comparatively rare in the population studied in the COPSAC_2010_ cohort, which could explain the absence of this association in that more comparably sized study.^64^

Several prior studies have reported higher levels of *Enterobacteriaceae* in colicky infants.^19,62,68,69^ However, we observed some evidence of this association only in our crude models (0–3 months, FDR = 0.07), and not in our covariate-adjusted models. This may suggest that the associations previously identified between *Enterobacteriaceae* and colic may have been confounded by one or more of the factors we included in our adjusted models. In our data, colic was negatively associated with *Bifidobacterium*, and some indirect evidence appeared to support the relevance of *Lactobacillus*, via principal components regression analyses. Lower *Lactobacillus* and lower *Bifidobacterium* have both been frequently reported in colicky infants.^8,11^ A prospective cohort with meconium samples found that the 19 infants who later developed colic had lower *Lactobacillus* than the 139 who did not.^10^ In a small but interesting longitudinal nested case-control study, de Weerth and colleagues,^62^ observed that the gut microbiota samples of 12 infants with colic at six weeks had lower levels of *Bifidobacterium* and *Lactobacillus* and were less diverse and less stable across repeated samples in the first month of life than those of 12 control infants. Furthermore, *Lactobacillus* has been repeatedly assessed in probiotic trials aimed at alleviating colic symptoms with some success, particularly with *L. reuteri*, now *Limosilactobacillus reuteri*.^11,13,17^ More recently *Bifidobacterium* probiotics have also been trialed in colic treatment. *B. lactis* (BB-12) reduced crying episodes and duration in two trials of colicky infants^16,70^ and a mix of *B. longum* and *Pediococcus pentosaceus* also reduced crying time.^71^

Trials of infant formula products supplemented with HMOs have sometimes included gastrointestinal symptoms or measurements as outcomes,^72^ including a trial of a mixture of five HMOs that increased stool frequency and softness.^73^ Puccio and colleagues found less colic and softer stools amongst C-section born infants given 2’-FL and LNnT supplemented formula.^74^ Another trial demonstrated a benefit of formula supplemented with galacto-oligosaccharides and long-chain fructo-oligosaccharides on colic incidence.^75^

The neutral HMOs LNH and LNnH, which we found to be associated with lower constipation risk amongst breastfed infants, do not appear to have been trialed as formula additives. Some prior work has speculated that HMOs convey some of the observed benefits of breastfeeding for infant gastrointestinal health.^75^ Speculatively, it is plausible that HMOs may influence gut motility, nociception, or inflammation through direct interactions with host receptors^76,77^ or through prebiotic modulation of microbiota.^42,78^ In our study, ever breastfeeding was associated with less constipation in the first three months, which is compatible with prior research finding generally higher defecation frequencies amongst breastfed infants.^33,79–81^

To contextualize our focus on the role of the gut microbiota on infant gastrointestinal health, we also report simple associations between other biopsychosocial risk factors and infant gastrointestinal symptoms. It was not an aim of this study to rigorously explore the full complexity of potential causal connections between all postulated biopsychosocial risk factors and infant gastrointestinal health. However, the breadth of associations identified in this work and prior studies highlights the importance of a holistic approach to understanding and managing infant and carer wellbeing, one which considers various biological, psychological, and social factors and their interactions. We observed consistent associations between maternal distress scores two weeks post-partum and the likelihood of the mother reporting persistent gastrointestinal symptoms in her infant. This included colic, constipation, and cramps symptoms throughout infancy. Links between maternal mental health and infant crying or colic symptoms have been reported in several previous studies, with maternal anxiety, distress, depression potentially acting both as cause and effect.^26,29^ Maternal history of IBS was strongly associated with report of infant cramps, but not colic or constipation, which may reflect a shared propensity for experiencing gastrointestinal discomfort, or a bias in maternal perception of this particularly subjective infant symptom. Prior work has suggested that functional gastrointestinal disorders such as IBS may have a genetic component.^82^ We found that maternal smoking during the third trimester associated with early constipation, but not with colic, although smoking during pregnancy has been associated with colic in prior work.^30,32,83,84^ Antibiotic exposure during pregnancy was also associated with more early infant constipation in our cohort, but not colic. One case-control study previously identified a link between intrapartum antibiotic exposure and colic.^85^ Antibiotic exposure during the first week of life was associated with colic risk in the INCA birth cohort study,^86^ which concurs with our observed association between antibiotic exposure in the first three months of life and colic during that period. Our data cannot rule out reverse causation for this association, but we were able to show that infant antibiotic exposure did not bias our observed microbiota associations, by repeating the analyses with only antibiotic-naïve infants. Stokholm and colleagues recently reported associations between infant colic and the presence of household pets, a positive association with cats and a negative association with dogs.^64^ We did not observe these associations, but did observe a positive association between dog ownership and infant constipation up to three months. However, we speculate that this is more likely explained by other biopsychosocial factors with which dog ownership was negatively correlated, including breastfeeding, maternal education, and maternal age.

Despite being the largest study yet of the gut microbiota and infant gastrointestinal health our observational design has several limitations. Maternal reporting of symptoms is subjective, and this likely biases associations between symptoms and psychosocial factors such as maternal distress score and parity. However, whilst this obfuscates the objective quantification of infant health state, the perceived severity of infant symptoms is clinically relevant in management of maternal wellbeing. In addition, by dichotomizing symptom persistence at three days per week, to delineate clinical significance, we potentially lose some relevant information on milder symptoms. Retrospective parental reporting of symptoms across three-month time windows introduces two limitations to our analyses. Firstly, imperfect recall may reduce our power to detect microbiota-symptom associations, and secondly, lack of fine temporal resolution prevents conclusions on the direction of causality for many of the associations that could be affected by early symptoms. To avoid the possibility of reverse causation where early gastrointestinal symptoms preceded feeding changes, we had to limit our classification of infant feeding status to never breastfed versus ever breastfed. Early symptoms may have also preceded the one-month microbiota sample.

Further large-scale studies are needed to clarify the role of the gut microbiota in colic and constipation etiology. Profiling of both fecal microbiota composition and gastrointestinal symptoms at multiple timepoints throughout infancy should help to unravel the direction of any causal relationships. To improve temporal and quantitative symptom detail and avoid parental recall bias, future studies could explore digital symptom reporting methods previously used in adult IBS studies, such as mobile diaries or experience sampling.^87^ As evidenced by various experimental models, plausible mechanistic links between infant microbes and pathophysiology of intestinal function can involve various microbial metabolites, including but not limited to, short and branched chain fatty acids, tryptophan metabolites, and secondary bile acids.^78,88^ Detailed *in vivo* profiling of microbiome functional capacity and activity through metagenomic, metatranscriptomic or metabolomic assays should then help to determine the relevance of experimentally plausible interactions to the physiology of real infants. Nonetheless, microbiome-focused research projects should not ignore the contextual relevance of maternal mental health, infant nutrition choices and timelines, and other relevant biopsychosocial factors.

## Supplementary Material

Supplemental Material

## Data Availability

The 16S rRNA gene amplicon sequencing data for this study have been deposited in the European Nucleotide Archive (ENA) at EMBL-EBI under the accession number PRJEB52836. https://www.ebi.ac.uk/ena/browser/view/PRJEB52836.
