# Supplementary material for: Why do babies cry? Exploring the role of the gut microbiota in infantile colic, constipation, and cramps in the KOALA birth cohort study
Source: Gut Microbes. 2025 Mar 30;17(1):2485326. doi: 10.1080/19490976.2025.2485326 (PMC11959906; doi:10.1080/19490976.2025.2485326)
Supplement: Supplemental Material [file KGMI_A_2485326_SM1545.zip › Supplment KGMI/20250216 clean copy KOALA microbiota GI manuscript.docx]

Supplementary figures


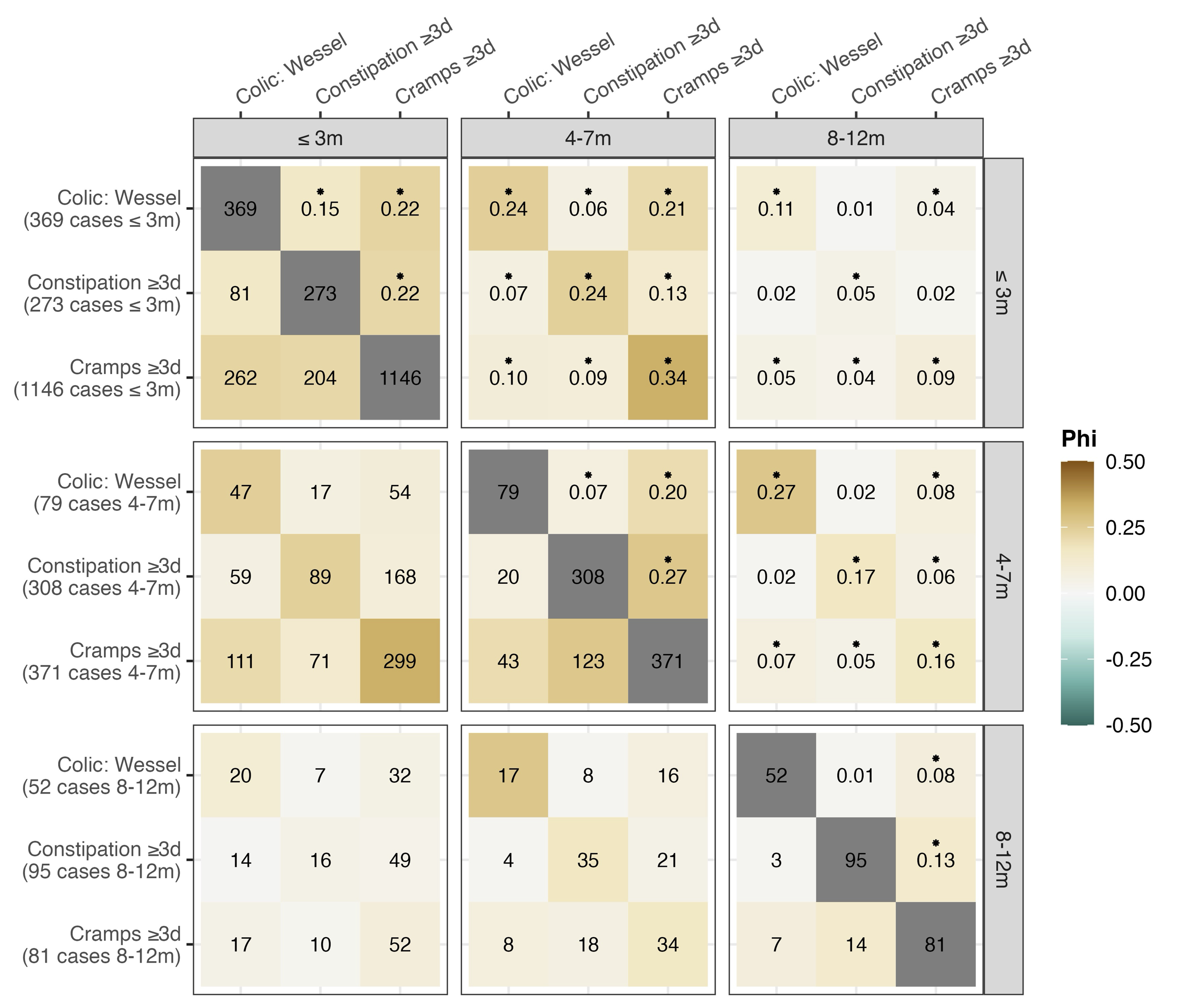


**Supplementary Figure 1 – Infant gastrointestinal symptom correlation analysis (N=2665)**Heatmap of Phi correlations (binary Pearson correlations) between each symptom at each timepoint, annotated with Phi correlation estimates (upper right half) and counts of children in whom the both symptoms occurred (lower left half). Counts on the diagonal are case numbers for that symptom, and the asterisk denotes p<0.05, without FDR correction.


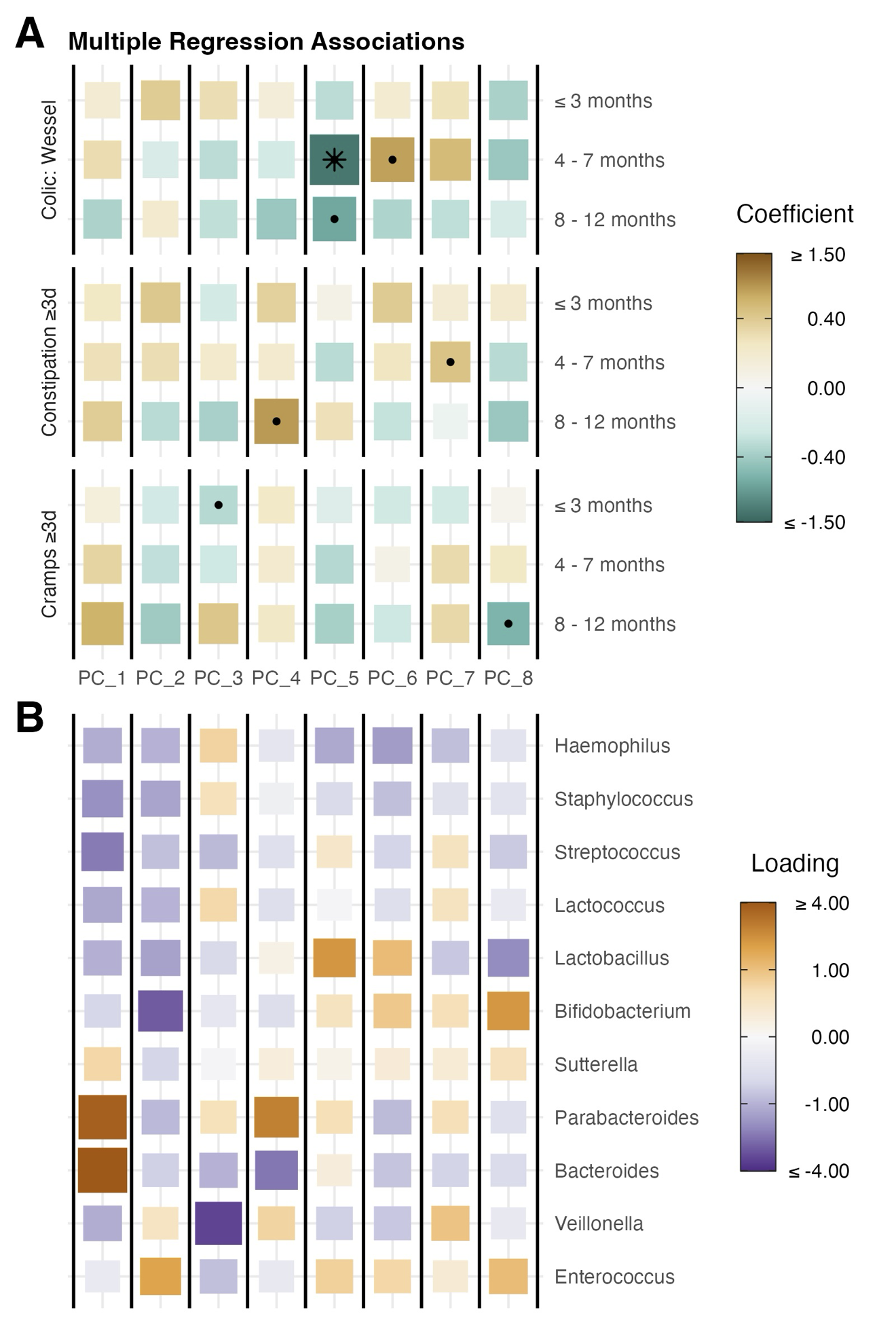


**Supplementary Figure 2 - Principal Components regression coefficients and PC loadings (N=1012)**A) Heatmap illustrating the direction and strength of associations between each PC and each gastrointestinal outcome. Dots indicate p<0.05 and asterisks indicate FDR<0.05 (corrected per outcome). Tile colour indicates the regression coefficient. Tile size is proportional to the absolute value of the regression coefficient.
B) Heatmap illustrating the loadings of each PC onto an influential subset of the original features, CLR-transformed genera. Tile size is proportional to the absolute value of the loading.
